# Aflatoxin B1 Disrupts Ovarian Follicular Dynamics and Activates Intrinsic Apoptosis‐Related Signaling in Rat Ovaries: Interactions Between Hormonal Imbalance and Follicular Atresia

**DOI:** 10.1155/bmri/5121993

**Published:** 2026-08-27

**Authors:** Farbod Poursadeghi, Mazdak Razi, Amir Amniattalab

**Affiliations:** ^1^ Department of Pathology, Faculty of Veterinary Medicine, Ur.C., Islamic Azad University, Urmia, Iran, azad.ac.ir; ^2^ Department of Basic Sciences, Division of Histology & Embryology, Faculty of Veterinary Medicine, Urmia University, Urmia, Iran, urmia.ac.ir

**Keywords:** aflatoxin B1, apoptosis, follicular growth, gonadotropin, ovary

## Abstract

**Background:**

Aflatoxin B1 (AFB1) is one of the most potent naturally occurring mycotoxins and has been implicated in reproductive toxicity. The present study is aimed at evaluating the time‐dependent effects of experimental AFB1 exposure on ovarian follicular dynamics, reproductive hormone profiles, and intrinsic apoptosis‐related markers in female rats.

**Methodology:**

Twenty‐four mature female Wistar rats were assigned to control and AFB1‐exposed groups (*N* = 6 per time point). AFB1 was administered intraperitoneally at 20 mg/kg body weight for 7, 14, and 21 days. Ovarian tissue and serum samples were collected at the end of each designated exposure period. Follicular population and atresia were evaluated histologically; serum concentrations of follicle‐stimulating hormone (FSH), luteinizing hormone (LH), estrogen, and progesterone were measured by ELISA, and the expression patterns of Bcl‐2, Bax, p53, and caspase‐3 were assessed using RT‐PCR and immunohistochemistry.

**Results:**

AFB1 exposure was associated with a time‐dependent reduction in total follicular population and a marked increase in follicular atresia. Serum FSH, LH, estrogen, and progesterone concentrations were significantly decreased in AFB1‐exposed animals compared with controls. At the molecular level, AFB1 decreased Bcl‐2 expression while increasing Bax, p53, and caspase‐3 expression in ovarian tissue, with prominent immunoreactivity in follicular cells.

**Conclusion:**

These findings indicate that experimental AFB1 exposure is associated with impaired follicular development, endocrine disruption, and activation of intrinsic apoptosis‐related signaling in rat ovaries. The observed coordinated changes suggest a biologically plausible interaction between hormonal imbalance and apoptosis‐associated pathways in AFB1‐induced follicular atresia, although functional validation is required to confirm direct causal mechanisms.

## 1. Introduction

In the tropical and subtropical regions, some species of fungi such as *Penicillium*, *Fusarium*, and *Aspergillus* grow in high temperatures and humidity [1]. Three molds of the *Aspergillus* species, including *Aspergillus flavus*, *Aspergillus parasiticus*, and *Aspergillus nomius*, are known to produce several types of aflatoxin (AF) [2]. Up to now, four common natural AFs, namely, AFB1, AFB2, AFG1, and AFG2, have been detected, with AFB1 being the most potent and toxic one among all of the mentioned types [3]. Indeed, AFB1, mostly by oral exposure and through contaminated food, enters the body [4]. Mechanistically, after entering the body, the AFB1 is metabolized in the liver by the P450 enzyme system into the ultimate carcinogen aflatoxin B1‐8,9‐epoxide (AFBO), which has two isomers, endo‐8,9‐epoxide and exo‐8,9‐epoxide [5]. The AFB1 and/or its metabolites may be found in agricultural foodstuffs such as peanuts, maize grains, cereals, milk, and milk products and have been shown to induce severe toxic symptoms in various kinds of animals, birds, fish, and even human individuals [6, 7]. Several reports (using animal models) have shown the AFB1‐induced deleterious effect on male and female reproductive capacity. Accordingly, previous studies on male animal models showed disruption of spermatogenesis, premature loss of spermatids, and decreased sperm count [8], enhanced sperm abnormality, and suppressed testicular steroidogenic enzyme contents as well as serum testosterone levels [9]. More recent findings showed that intraperitoneal administration of 20 *μ*g/kg body weight from AFB1 in a mouse model suppresses the testicular antioxidant status, triggers the mitochondria‐dependent apoptosis pathway, and negatively affects the cell cycle machinery at the germ cell level [3, 6]. In females, Mahady et al. showed that 250, 500, and 1000 ppb AFB1/kg diet in Albino rats significantly suppressed the estrous cycle; diminished the serum estradiol, progesterone, and testosterone levels; significantly increased the follicular atresia; and elevated the number of inactive corpus luteums in the ovaries [10]. Other animal studies showed that chronic and subchronic exposure to AFB1 reduces the ovarian and uterine sizes, downregulates the follicular growth ratio in the ovaries, increases fetal resorption, and results in implantation loss and intrauterine death [1, 11]. El‐Azab et al. investigated the presence of AFB1 in sera of infertile women and showed the correlation between AFB1 and ovarian disorders and hormonal impairments, indicating the AFB1‐induced negative effect on female infertility even in the human population [12]. In addition to classical apoptosis, AFB1 exposure can trigger complex cell death modalities. Pterostilbene supplementation was recently reported to inhibit PANoptosis—a combined form of apoptosis, necroptosis, and pyroptosis—in mouse ovaries, normalizing ovarian gene expression and preserving follicular architecture and hormonal balance [13]. Apoptosis is initiated through two principal “extrinsic” and “intrinsic” pathways. The extrinsic pathway is mainly mediated by the subgroup of the TNF receptor superfamily. However, the intrinsic pathway is controlled by members of the B‐cell lymphoma‐2 (Bcl‐2) family and is possibly referred to as the Bcl‐2‐controlled pathway [8]. Indeed, the Bcl‐2 family consists of several members, such as proapoptotic (e.g., Bax and Bak) and antiapoptotic (e.g., Bcl‐2, Bcl‐XL, and Mcl‐1) genes, which are known as key regulators of intrinsic apoptosis. As an antiapoptotic protein, Bcl‐2 prevents the Bax (proapoptotic protein) oligomerization and/or binds with Bax, making Bcl‐2/Bax complex, and via these mechanisms, Bcl‐2 inactivates Bax, which in turn results in the imprisonment of cytochrome c in mitochondria. Otherwise, the suppressed expression of Bcl‐2 evokes Bax oligomerization, leading to the release of several apoptogenic molecules such as cytochrome c and caspase family activation [6, 14, 15]. Aside from the role of Bcl‐2 in intrinsic apoptosis, the role of tumor suppressor p53, as another participating gene in the intrinsic apoptosis pathway, should not be forgotten. Accordingly, p53 overexpression after severe DNA damage initiates apoptosis, DNA repair at G1/S, and/or cell cycle arrest at the G2/M stage [16, 17]. To make a cross‐link between p53 and mitochondria‐dependent apoptosis, one should note that p53 binds to Bcl‐XL and evokes the puma overexpression, resulting in Bax overexpression, conformation, and translocation to the mitochondria [16, 18]. Next, the Bax translocation/oligomerization diminishes the mitochondrial membrane potential of mitochondrial, which in turn results in cytochrome c release and triggers DNA fragmentation and degradation of cytoskeletal and nuclear proteins through caspase‐dependent pathways [6].

Although AFB1‐related reproductive toxicity has been documented in both male and female experimental models, comparatively limited information is available regarding its coordinated effects on ovarian follicular dynamics, reproductive endocrine status, and intrinsic apoptosis‐associated molecular markers. Ovarian follicular survival depends on a tightly regulated interaction between gonadotropin support, steroid hormone production, granulosa cell integrity, and apoptosis‐related signaling. Therefore, evaluating hormonal changes together with follicular morphology and the expression of Bcl‐2 family members, p53, and caspase‐3 can provide a more integrated view of AFB1‐associated ovarian injury. In this context, the present study was designed to investigate the time‐dependent effects of experimental AFB1 exposure on follicular growth and atresia, serum follicle‐stimulating hormone (FSH), luteinizing hormone (LH), estrogen, and progesterone concentrations and ovarian expression of Bcl‐2, Bax, p53, and caspase‐3. The novelty of the study lies in linking histological, endocrine, transcriptional, and immunohistochemical (IHC) endpoints to characterize the coordinated ovarian response to AFB1 exposure while interpreting the findings within the limits of the applied assays.

While previous studies have primarily focused on isolated aspects of the AFB1‐induced female reproductive toxicity mechanism, such as hormonal deregulation, altered follicular dynamics, or ovarian atresia, the contribution of the present study is the parallel assessment of follicular‐stage distribution and ovarian atresia, combined with serum gonadotropin and steroid hormone levels and semiquantitative transcript alterations, as well as the ovarian follicular localization of apoptosis‐associated immunoreactivity. These findings provide evidence for a complex interplay of endocrine, follicular, and molecular mechanisms in AFB1‐induced ovarian toxicity without directly establishing causal relationships.

## 2. Materials and Methods

### 2.1. Chemicals

AFB1 was purchased from Sigma CO (Sigma, St. Louis, Missouri). The rabbit anti‐rat/mouse primary antibodies for Bcl‐2 (Elabscience, United States, Cat No. E‐AB‐60788), p53 (Elabscience, United States, Cat No. E‐AB‐32472), Bax (Elabscience, United States, Cat No. E‐AB‐22128), and caspase‐3 (Elabscience, United States, Cat No: E‐AB‐63510) were obtained from Life‐Tebgen Co. All other chemicals, which were used, were commercial products of analytic grade.

### 2.2. Animals and Grouping

The current study was approved by the Research Committee of the Faculty of Veterinary Medicine, Islamic Azad University, Urmia Branch (Reg. No. 1708984). Twenty‐four mature female Wistar rats were obtained from the Laboratory Animal Center, Faculty of Veterinary Medicine, Urmia University, Urmia, Iran. Animals were maintained under standard laboratory conditions with free access to food and water and were allowed to acclimatize before the experiment. The rats were assigned to the control and AFB1‐exposed groups (*N* = 6 per time point). AFB1 was prepared essentially according to the previously described experimental toxicology protocol of Faisal et al. [8]. Briefly, AFB1 was dissolved in corn oil and ethanol (95:5, vol/vol) and administered intraperitoneally at 20 mg/kg body weight for the designated exposure periods of 7, 14, and 21 days. Control animals received the vehicle alone. The selected dose and route were used as a controlled experimental exposure paradigm to induce measurable ovarian toxicological responses and to enable time‐dependent assessment of histological, hormonal, and apoptosis‐related alterations. During the experiment, animals were monitored daily for general appearance, activity, food and water intake, mobility, and visible signs of distress. No procedure‐related mortality was observed during the experimental period. All procedures were conducted in accordance with the ethical approval granted by the Animal Model Research Committee of Islamic Azad University, Urmia Branch.

The selected exposure protocol should be regarded as an isolated experiment; therefore, the results should not be extrapolated to chronic dietary exposure. The route of administration significantly impacts the way of absorption, and therefore, distribution, metabolism, and toxicity. In this regard, although intraperitoneal administration can be useful for understanding the mechanism of toxicity, it should not be used for quantification of dietary exposure.

### 2.3. Histological Analysis

Following 7, 14, and 21 days, the ovaries were dissected out, fixed in 10% formalin and processed and embedded in paraffin. The specimens were then cut serially by a rotary microtome (LKB, United Kingdom) and stained with the hematoxylin and eosin technique. The ovarian follicles were characterized in terms of their stage as primordial, primary, secondary, tertiary, and graafian, and their morphology was examined by light microscope (under 100× magnification). The follicles with a complete/normal layer of intact and flattened granulosa cells and a normal oocyte were considered normal follicles. However, those with granulosa cell dissociation, early antrum formation, early granulosa cell luteinization, atrophied oocyte, and oocyte elimination were considered atretic follicles. The follicular count was performed by counting follicles in all slides, and finally, the total follicle count per ovary and atretic follicles relative to total follicles were examined and compared between groups.

### 2.4. IHC Staining

The IHC staining protocol was carried out based on the methods used in our previously established protocols, with minor modifications [6]. For this purpose, the paraffin sections (5–6 *μ*m) of ovarian tissue were prepared, deparaffinized, and rehydrated. Next, the histological slides were incubated with 0.3% H_2_O_2_ (10 min) to block endogenous peroxidase activity. Then, the slides were washed with phosphate‐buffered saline (PBS, pH 7.4), incubated with 5% bovine serum albumin (BSA) in PBS for 20 min. Then, the slides were incubated with anti‐Bcl‐2 (1:100), anti‐Bax (1:200), anti‐p53 (1:150), and anti‐caspase‐3 (1:100) antibodies (at 4°C overnight). Next, the histological sections were rinsed in PBS and incubated in the secondary antibody (HRP‐conjugated goat anti‐rabbit; CWBIO, Beijing, China) at 37°C for 30 min, washed with PBS again, and incubated in the presence of 3,3‐diaminobenzidine (DAB) substrate. Finally, sections were counterstained with hematoxylin and dehydrated and mounted with neutral balsam. The positive immunoreactivity was marked as brown. The percentage of follicles with positive immunoreactivity in more than 50% of follicular cells and the number of immunoreactive cells per square millimeter of tissue were analyzed and compared between groups.

### 2.5. Serum Sampling and Hormonal Assay

At the end of each designated experimental period, namely, 7, 14, and 21 days after AFB1 administration, the animals were anesthetized, and blood samples were collected directly from the heart. The samples were centrifuged at 3000 rpm for 5 min to separate serum. The collected serum samples were stored at −70°C until analysis. Serum concentrations of FSH, LH, estrogen, and progesterone were measured using ELISA kits according to the manufacturers′ instructions and compared between groups. Serum FSH and LH levels were evaluated using commercial kits (MyBioSource, United States; FSH: MBS263261 and LH: MBS764675). Serum progesterone and estrogen levels were analyzed using commercial kits (Progesterone: Novus Biologicals, Canada, Cat No. NBP2‐60127; estrogen: MyBioSource, United States, Cat No. MBS766177).

### 2.6. RNA Extraction, cDNA Synthesis, and Reverse Transcription‐PCR (RT‐PCR)

Total RNA was extracted from the rat ovarian tissue samples using TRIzol reagent (Invitrogen; Thermo Fisher Scientific Inc.), and thereafter, the RNA concentration and quality (at 260 nm and A260/280 = 1.8–2.0) were evaluated by spectrophotometry (Thermo Scientific, Washington, United States). Next, first‐strand cDNA was synthesized using a cDNA synthesis kit (Pars Tous Biotechnology, Iran, Cat No A101161) according to the manufacturer′s protocol. Subsequently, reverse transcription was carried out using PCR Master Mix (Biofact, Korea, Cat No. ST302‐50 h) following the manufacturer′s instructions. The PCR conditions were run as follows: general denaturation at 95°C for 3 min, one cycle, followed by 35 cycles of 94°C for 20 s; annealing temperature (52°C for 1 min for p53, 62°C for 1 min for Bcl‐2, 50°C for 30 s for caspase‐3, and 63°C for 30 s for GAPDH); and elongation: 72°C for 1 min and 72°C for 5 min. Specific primer sequences are presented in Table 1 [15, 19, 20]. The amplified products were electrophoresed in 1.5% agarose gels, stained with ethidium bromide, and viewed using an ultraviolet (UV) Trans‐illuminator (ATP technology, Iran). The ethidium bromide–stained gel bands were scanned, and the signal intensities were quantified by the computerized Gel‐Pro analysis software (ATP, Version 2.1 for Windows 7) and normalized relative to GAPDH (internal control).

**Table 1:** The primers′ details.

Target genes	Primers (5 ^′^‐3 ^′^)
Bcl‐2	FWD: CTGGTGGACAACATCGCTCTG
	RVS: GGTCTGCTGACCTCACTTGTG
Bax	FWD: AAGAAGCTGAGCGAGTGTCT
	RVS: CAAAGATGGTCACTGTCTGC
P53	FWD: ATGGAGGAGTCACAGTCGGATA
	RVS: GACTTCTTGTAGATGGCCATGG
Caspase‐3	FWD: TACCCTGAAATGGGCTTGTGT
	RVS: GTTAACACGAGTGAGGATGTG
GAPDH	FWD: CAAATTCAACGGCACAGTCA
	RVS: ACACATTGGGGGTAGGAACA

Because the products were measured after the PCR amplification was complete, it was classified as a semiquantitative procedure. The values are derived from the density measurements and represent target‐to‐GAPDH band intensity ratios and should not be considered accurate or precise transcript abundance values.

### 2.7. Statistical Analysis

Photomicrographs were taken using a Canon onboard camera (Japan, PC1587). Figures were merged and prepared using Adobe Photoshop CS10 (Adobe Systems Inc., Mountain View, California, United States). Quantitative histological and IHC evaluations were performed in a blinded manner. The Kolmogorov–Smirnov and Levene′s tests were used to assess data normality and homogeneity of variance, respectively. All quantitative data are presented as mean ± SD (*N* = 6 rats in each group) and were statistically analyzed using one‐way ANOVA followed by appropriate post hoc tests. Differences were considered statistically significant at *p* < 0.05.

The experimental design consisted of four treatment groups, of which one was the vehicle‐control and the others were exposed to AFB1, with the exposure duration being 7, 14, or 21 days, respectively. Since in the current design separate vehicle‐control groups were not used for each time point, treatment and time variables were not fully crossed, and thus, the interaction between treatment and time could not be evaluated. Therefore, for the analysis of this experiment, a one‐way ANOVA was used across four treatment groups as defined above.

## 3. Results

### 3.1. AFB1 Significantly Decreased the Follicular Population in the Ovary and Increased Atresia

**Figure 1:**
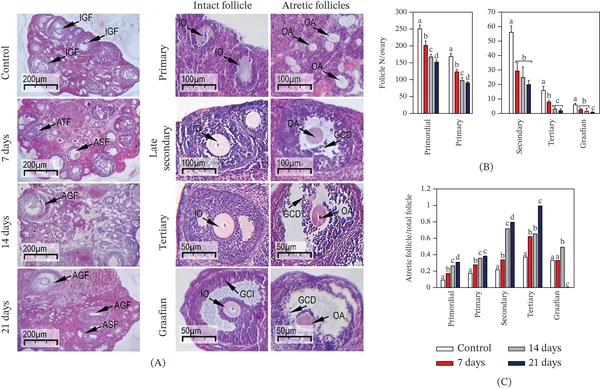
AFB1‐induced damages on follicular population and atresia. (A) Cross‐sections of ovarian tissue in different groups. Note normal follicles at different stages in the control group. However, the sections from different days after exposure to aflatoxin B1 (AFB1) present severe follicular atresia at various stages. (B) Mean total follicle numbers *per* ovary in different groups at different stages and (C) relative number of atretic follicles to total follicles *per* ovary in different groups. All data are presented as mean ± SD (*N* = 6 rats in each group), and *p* < 0.05 was considered statistically different. Different letters indicate significant differences between groups. IGF, intact graafian follicle; AGF, atretic graafian follicle; ASF, atretic secondary follicle; IO, intact oocyte; OA, oocyte atresia; GCI, granulosa cell integrity; GCD, granulosa cell dissociation.

The time‐dependent effect of AFB1 against follicular growth was analyzed by counting the follicles at different primordial, primary, secondary, tertiary, and graafian stages. Observations showed that AFB1, in a time‐dependent manner, decreased the follicular population in the ovaries. Severe granulosa cell dissociation, oocyte elimination/atrophy, nuclear pyknosis, and cumulus cell dissociation were revealed on AFB1‐treated ovaries (Figure 1A,B). Moreover, to find out the effect of AFB1 on follicular atresia, the relative ratio of atretic follicles per total follicles in the ovary was estimated. Observations revealed that AFB1, time‐dependently, increased the atresia ratio compared to the control animals (Figure 1C).

### 3.2. AFB1 Diminished the Serum Levels of Gonadotropins and Ovarian Hormones

**Figure 2:**
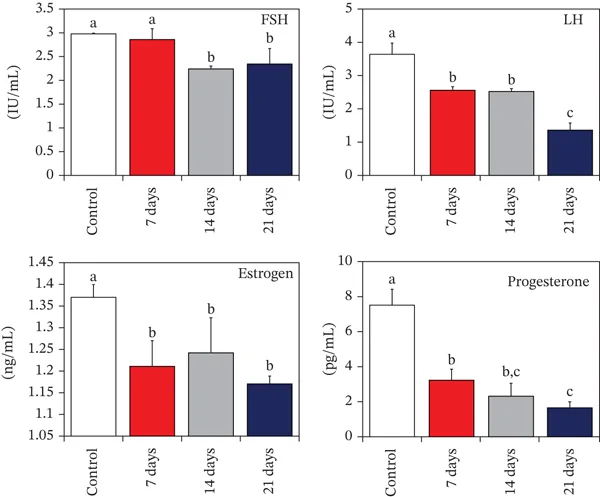
Time‐dependent effect of aflatoxin B1 (AFB1) on serum levels of gonadotropins and ovarian source hormones. All data are presented as mean ± SD (*N* = 6 rats in each group), and *p* < 0.05 was considered statistically different. Different letters indicate significant differences between groups.

To analyze the possible time‐dependent effect of AFB1 on the hypophyseal–ovarian axis, the serum levels of LH, FSH, estrogen, and progesterone were evaluated and compared between groups. Biochemical analyses showed a significant (*p* < 0.05) reduction in serum levels of LH and FSH, which developed time‐dependently. Moreover, the animals in AFB1‐treated groups exhibited a remarkable (*p* < 0.05) reduction in serum estrogen and progesterone levels (Figure 2).

### 3.3. AFB1 Suppressed the Bcl‐2 Expression Time‐Dependently

**Figure 3:**
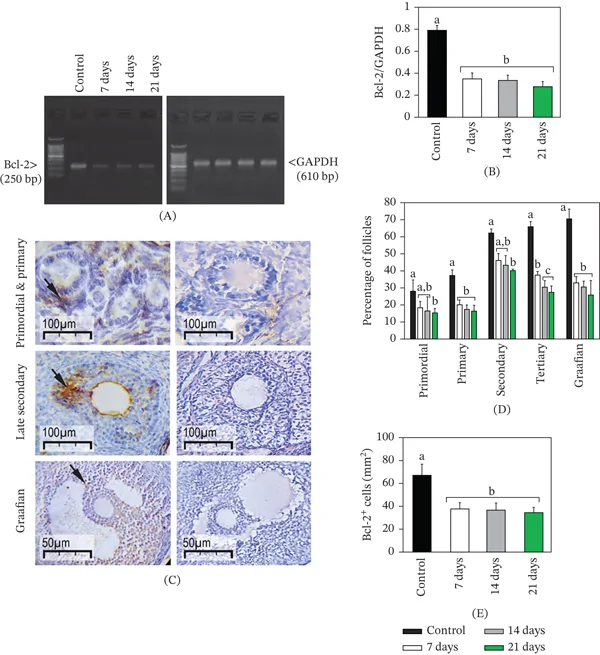
Time‐dependent effect of aflatoxin B1 (AFB1) on the expression level of Bcl‐2. (A) RT‐PCR gel electrophoresis for Bcl‐2 mRNA, (B) relative expression of Bcl‐2 to GAPDH in different groups, (C) mean percentage of follicles with positive reaction for Bcl‐2 in different groups, and (D) Bcl‐2^+^ cell number *per* square millimeter of tissue in different groups. All data are presented as mean ± SD (*N* = 6 rats in each group), and *p* < 0.05 was considered statistically different. Different letters indicate significant differences between groups. Note: The positive reaction for Bcl‐2 is marked with arrows.

The effect of AFB1 on Bcl‐2 expression was investigated by RT‐PCR and IHC staining. The RT‐PCR analysis showed that AFB1 significantly (*p* < 0.05) decreased the Bcl‐2 mRNA level, time‐dependently (Figure 3A,B). Accordingly, the animals in the AFB1‐treated groups showed the lowest mRNA level after 21 days versus the control group. Next, to see the effect of AFB1 on protein content, the percentage of follicles (solely) that represented Bcl‐2 was evaluated in each group and compared between groups. Finally, to reduce histological errors, the number of Bcl‐2^+^ *per* square millimeter of tissue was analyzed. Light microscopic analyses revealed that AFB1 significantly (*p* < 0.05) diminished the percentages of Bcl‐2^+^ follicles compared to the control animals. This situation developed time‐dependently (Figure 3C,D). Moreover, the number of Bcl‐2^+^ cells *per* square millimeter of ovarian tissue was decreased time‐dependently in AFB1‐treated animals (Figure 3E).

### 3.4. AFB1 Increased the Bax Expression Time‐Dependently

**Figure 4:**
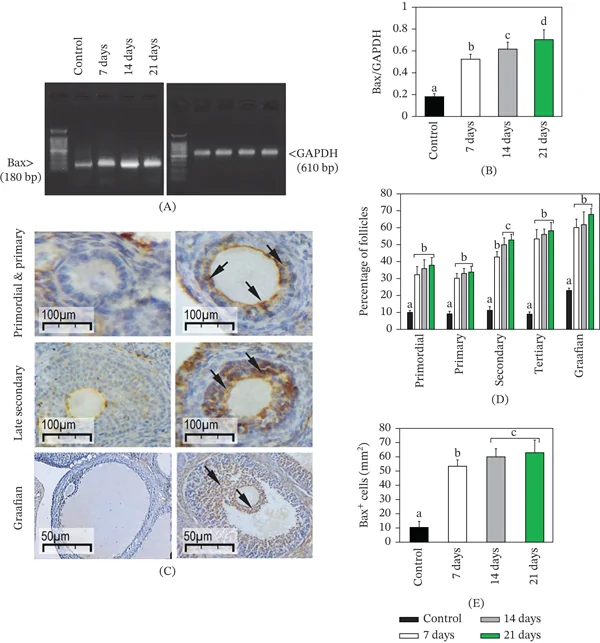
Time‐dependent effect of aflatoxin B1 (AFB1) on the expression level of Bax. (A) RT‐PCR gel electrophoresis for Bax mRNA, (B) relative expression of Bax to GAPDH in different groups, (C) mean percentage of follicles with positive reaction for Bax in different groups, and (D) Bax^+^ cell number *per* square millimeter of tissue in different groups. All data are presented as mean ± SD (*N* = 6 rats in each group), and *p* < 0.05 was considered statistically different. Different letters indicate significant differences between groups. Note: The positive reaction for Bax is marked with arrows.

Similar to Bcl‐2, the effect of AFB1 on Bax expression was investigated by RT‐PCR and IHC staining. The RT‐PCR analysis showed that AFB1 significantly (*p* < 0.05) upregulated the Bax mRNA level, time‐dependently (Figure 4A,B). In continuation, to see the effect of AFB1 on protein content, the percentage of follicles, which represented Bax, was evaluated in each group and compared between groups. Observations revealed a significant increment in the percentage of follicles with positive reaction for Bax, time‐dependently (*p* < 0.05). The Bax^+^ reactions were revealed mostly in granulosa cells (Figure 4C,D). Finally, to reduce histological errors, the number of Bax^+^ per square millimeter of tissue was analyzed. The number of Bax^+^ cells *per* square millimeter of ovarian tissue was increased time‐dependently in AFB1‐treated animals (Figure 4E).

### 3.5. AFB1, Time‐Dependently, Increased the p53 Expression

**Figure 5:**
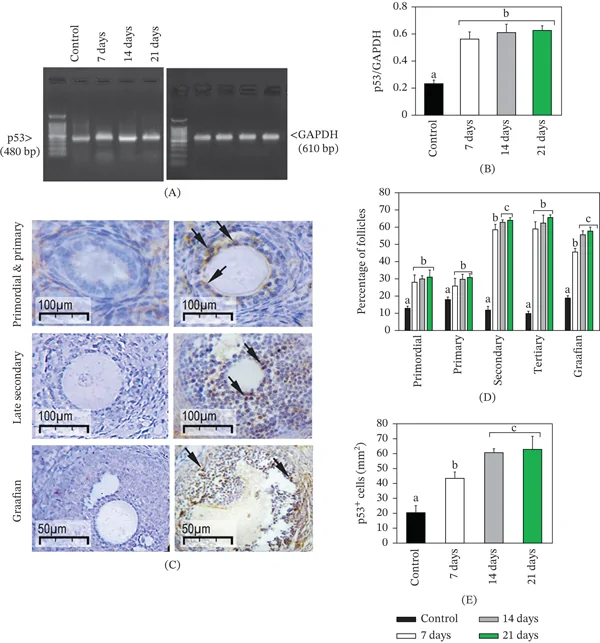
Time‐dependent effect of aflatoxin B1 (AFB1) on the expression level of p53. (A) RT‐PCR gel electrophoresis for p53 mRNA, (B) relative expression of p53 to GAPDH in different groups, (C) mean percentage of follicles with positive reaction for Bax in different groups, and (D) p53^+^ cell number *per* square millimeter of tissue in different groups. All data are presented as mean ± SD (*N* = 6 rats in each group), and *p* < 0.05 was considered statistically different. Different letters indicate significant differences between groups. Note: The positive reaction for p53 is marked with arrows.

The animals in the AFB1‐treated groups represented a remarkable (*p* < 0.05) increment in the p53 mRNA level versus the control animals. Accordingly, the animals in the 21‐day AFB1‐treated group represented the highest p53 mRNA level (Figure 5A,B). Thereafter, the IHC‐stained slides were analyzed, and the cross‐sections of AFB1‐treated groups represented a significant (*p* < 0.05) increment in the percentage of follicles with p53^+^ reaction compared to the control group. This situation was developed time‐dependently (Figure 5C,D). Finally, the increasing number of p53^+^ cells per square millimeter of ovarian tissue was revealed in the AFB1‐treated groups (Figure 5E).

### 3.6. AFB1 Significantly Upregulated the Caspase‐3 Expression, Time‐Dependently

**Figure 6:**
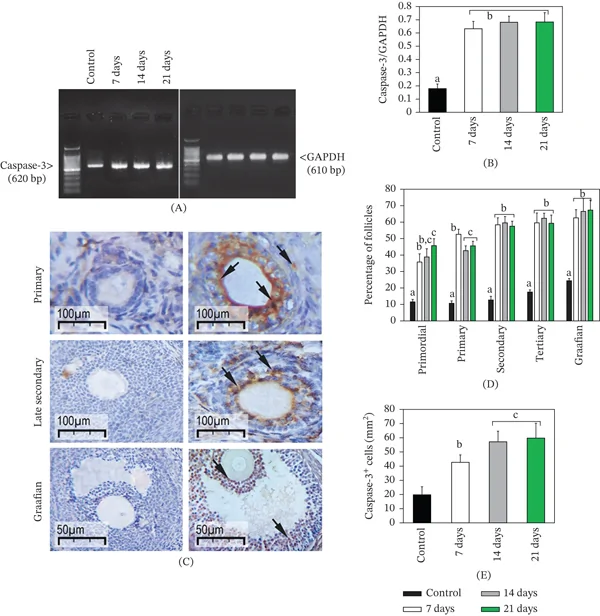
Time‐dependent effect of aflatoxin B1 (AFB1) on the expression level of caspase‐3. (A) RT‐PCR gel electrophoresis for caspase‐3 mRNA, (B) relative expression of caspase‐3 to GAPDH in different groups, (C) mean percentage of follicles with positive reaction for caspase‐3 in different groups, and (D) caspase‐3^+^ cell number *per* square millimeter of tissue in different groups. All data are presented as mean ± SD (*N* = 6 rats in each group), and *p* < 0.05 was considered statistically different. Different letters indicate significant differences between groups. Note: The positive reaction for caspase‐3 is marked with arrows.

The RT‐PCR analysis showed a significant (*p* < 0.05) increment in caspase‐3 mRNA level in AFB1‐treated animals compared with the control group. Although caspase‐3 mRNA expression was increased in all AFB1‐exposed groups, no significant differences were detected among the AFB1‐treated time points (Figure 6A,B). IHC staining demonstrated a remarkable (*p* < 0.05) increase in the percentage of follicles with caspase‐3+ immunoreactivity in AFB1‐treated animals compared with controls (Figure 6C,D). Finally, the number of caspase‐3+ cells per square millimeter of ovarian tissue was elevated in the AFB1‐exposed groups (Figure 6E), indicating that AFB1 exposure was associated with enhanced caspase‐3‐related immunoreactivity in ovarian follicular cells.

## 4. Discussion

The present study investigated the time‐dependent effects of experimental AFB1 exposure on ovarian follicular dynamics, reproductive hormone status, and intrinsic apoptosis‐related markers in female rats. The principal findings were that AFB1 exposure was associated with increased follicular atresia; reduced total follicular population; decreased serum FSH, LH, estrogen, and progesterone levels; downregulation of the antiapoptotic marker Bcl‐2; and increased expression of Bax, p53, and caspase‐3. These results collectively indicate that AFB1‐induced ovarian injury is accompanied by coordinated endocrine disruption and apoptosis‐associated molecular alterations. Importantly, the revised interpretation avoids assigning a definitive causal sequence and instead emphasizes the association between the measured histological, hormonal, transcriptional, and IHC endpoints.

The main value of this study is the association of the findings related to ovarian morphology and endocrine status with transcript‐level data and cell‐localized immunoreactivity. The decreases in the number of follicles and the increases in follicular atresia observed upon AFB1 exposure were linked to the suppression of gonadotropins and ovarian steroid hormones as well as a switch in the balance between anti‐ and proapoptotic signaling. The fact that similar trends were observed within the same set of experiments allows a more consistent interpretation of the mechanisms underlying AFB1‐induced ovarian damage than would be possible by separately reviewing the results. However, this does not necessarily indicate that the changes in ovarian hormonal environment are responsible for the increases in apoptotic signaling and follicular atresia.

The reduction in gonadotropins and ovarian steroid hormones observed in the present study is consistent with previous reports describing AFB1‐associated impairment of reproductive endocrine function in female animals and reproductive disorders in exposed populations [10, 12, 21]. FSH and LH are essential for follicular recruitment, granulosa cell function, steroidogenesis, follicular maturation, and ovulatory competence. Therefore, the observed decrease in FSH and LH, together with reduced estrogen and progesterone levels, provides an endocrine context for the impaired follicular growth and increased atresia detected histologically. However, because hormonal rescue or receptor‐level analyses were not performed, the relationship between gonadotropin suppression and apoptosis‐related marker expression should be interpreted as a biologically plausible association rather than direct proof of endocrine‐driven apoptosis. This more cautious interpretation is also supported by the concurrent reduction in follicular population and the increase in atretic follicles across the AFB1‐exposed groups.

An important source of biological variability in the present study is the stage of the estrous cycle at the time of sacrifice. Proestrus, estrus, metestrus, and diestrus in rats are characterized by distinct fluctuations in gonadotropin levels, ovarian hormone concentrations, follicular recruitment, ovulation, luteinization, and follicular atresia. Since vaginal cytology was not performed and animals were not staged to have a consistent estrous phase at the time of sacrifice, the variance between groups may be partially explained by variations in estrous stage producing differences in ovarian morphology and serum hormone concentrations. Thus, although AFB1 treatment produced consistent histopathological, endocrine, and molecular responses across experiments, estrous variability may have contributed to some of the observed effects. Subsequent experiments should include daily vaginal smear analysis and collection of tissue and serum at comparable estrous stages.

At the molecular level, the simultaneous decrease in Bcl‐2 and increase in Bax, p53, and caspase‐3 indicate that AFB1 exposure was associated with activation of intrinsic apoptosis‐related signaling in ovarian tissue. The Bcl‐2/Bax balance is an important determinant of mitochondrial membrane integrity and cellular susceptibility to apoptosis, whereas p53 can participate in stress‐related transcriptional regulation of proapoptotic pathways [16, 18, 22]. In the present study, increased Bax and p53 expressions, together with reduced Bcl‐2 expression, suggest a shift toward a proapoptotic profile in AFB1‐exposed ovaries. Nevertheless, because cytochrome c release, cleaved caspase‐3, TUNEL staining, or Western blot confirmation were not performed, the findings should be described as evidence of altered apoptosis‐associated signaling rather than definitive functional confirmation of apoptosis execution.

Although the reduction of Bcl‐2 and the increase of Bax, p53, and caspase‐3 suggest a shift toward the implementation of the apoptosis mechanism, these data are indirect evidence of the process. The current research did not include the evaluation of mitochondrial outer membrane permeability, cytochrome c release, caspase‐3 enzyme activity, DNA fragmentation, and the abundance of apoptosis‐related proteins. In addition, the presented results of cleaved caspase‐3 immunostaining do not confirm the presence and the role of this enzyme in the development of apoptosis. Thus, the changes at the transcriptional level and the presence of activated caspase‐3 should be considered indirect evidence of the ongoing process. Further studies analyzing TUNEL positivity, active form of cleaved caspase‐3, cytochrome c content, enzyme activity assays, and the application of pharmacological inhibitors of apoptosis pathways are needed to prove the role of the intrinsic pathway in AFB1‐induced follicular injury.

**Figure 7:**
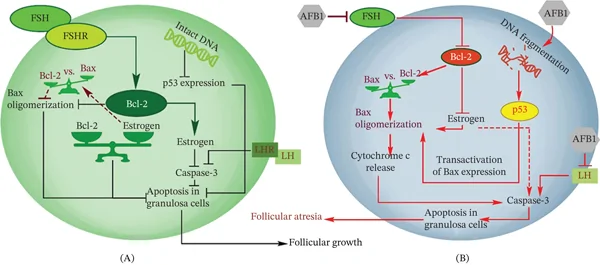
Schematic representation of the proposed interactions among reproductive hormones, follicular dynamics, and intrinsic apoptosis‐related markers under physiological and AFB1‐exposed conditions. (A) Under physiological conditions, FSH and LH support follicular development, steroidogenesis, granulosa cell survival, and maintenance of the Bcl‐2/Bax balance. (B) In the AFB1‐exposed condition, the present findings indicate reduced serum FSH, LH, estrogen, and progesterone levels, decreased Bcl‐2 expression, and increased Bax, p53, and caspase‐3 expression. These coordinated changes may contribute to granulosa cell injury, follicular degeneration, and follicular atresia. The schematic is intended to summarize biologically plausible associations based on the measured endpoints and should not be interpreted as definitive proof of a direct causal sequence.

The increase in caspase‐3 expression and immunoreactivity further supports the involvement of apoptosis‐associated pathways in follicular damage following AFB1 exposure. Caspase‐3 is commonly regarded as a key executioner molecule in apoptotic signaling, and increased caspase‐3 expression in granulosa cells has been linked to follicular atresia in several reproductive models [23, 24]. In the present study, caspase‐3 immunoreactivity was observed in follicular cells, particularly in association with atretic follicles, which aligns with the histological evidence of granulosa cell dissociation, oocyte atrophy, and follicular degeneration. Thus, the combined histological, hormonal, RT‐PCR, and IHC findings support the interpretation that experimental AFB1 exposure is associated with ovarian endocrine disturbance and apoptosis‐related follicular injury. A schematic summary of the proposed associations among hormonal suppression, follicular degeneration, and apoptosis‐related signaling under AFB1 exposure is presented in Figure 7. The present study used an experimental AFB1 challenge model to evaluate controlled time‐dependent ovarian responses; therefore, the selected dose and intraperitoneal route should be interpreted as a toxicological exposure paradigm rather than a direct simulation of chronic dietary exposure. Future studies using lower oral doses, longer exposure periods, estrous cycle staging, and functional validation assays will be valuable for translating these findings to physiologically relevant exposure scenarios.

Under natural conditions, the exposure of AFB1 usually occurs by swallowing contaminated food or feed, which involves both repeated exposure to the toxin and relatively small doses, as compared to the experimental challenge models. On the other hand, the oral and intraperitoneal routes of administration may differ substantially in terms of gastrointestinal physiology and first‐pass metabolism, oral bioavailability, peak exposure, and distribution of the toxicant in bodily tissues. Thus, the results of the current study should be further evaluated in the context of hazard characterization and mechanism‐driven research, while the obtained data on reproductive toxicity cannot be extrapolated into a quantitative risk assessment of prolonged exposure at low doses in humans or animals. Therefore, the impact of AFB1 on reproduction may need further investigation through repeated oral dose experiments combined with lengthy observations and the measurement of internal exposure biomarkers.

## 5. Conclusion

In conclusion, the present study demonstrates that experimental AFB1 exposure is associated with time‐dependent impairment of ovarian follicular dynamics; increased follicular atresia; reduced serum LH, FSH, estrogen, and progesterone levels; and altered expression of intrinsic apoptosis‐related markers, including decreased Bcl‐2 and increased Bax, p53, and caspase‐3. These findings suggest that AFB1‐induced ovarian injury may involve coordinated endocrine disruption and activation of apoptosis‐associated signaling in follicular cells. However, because the study did not include functional pathway inhibition, hormonal rescue, estrous synchronization, or direct oxidative stress assays, the proposed interactions should be interpreted as biologically plausible associations rather than definitive causal mechanisms.

### 5.1. Limitations

The present study has several limitations that should be considered when interpreting the findings. First, the experimental AFB1 exposure model was designed to induce measurable ovarian toxicological responses under controlled conditions and does not fully reproduce chronic low‐dose dietary exposure. Second, oxidative stress and antioxidant defense markers were not directly measured; therefore, oxidative stress is discussed only in relation to previous literature and not as a direct outcome of the present study. Third, estrous cycle monitoring or synchronization was not performed, which may influence ovarian histology and circulating reproductive hormone levels. Fourth, although RT‐PCR and IHC analyses demonstrated coordinated changes in Bcl‐2, Bax, p53, and caspase‐3, additional assays such as Western blotting, cleaved caspase‐3 detection, TUNEL staining, hormonal rescue, or pathway inhibition would be valuable for confirming functional apoptosis and causal mechanisms. Future studies addressing these aspects will further clarify the reproductive toxicity of AFB1 under physiologically relevant exposure conditions.

Furthermore, conventional endpoint RT‐PCR with gel densitometry is semiquantitative, and quantification may lack full reliability due to factors including different amplification efficiencies, cycle threshold and plateau effects, EtBr staining and autoradiography, gel‐scanning normalization, and saturation. Therefore, quantitative validation using real‐time PCR with efficiency‐corrected primers and multiple reference genes is required. Moreover, without an independent vehicle control, it cannot be excluded that exposure duration interacts with the treatment effect. Therefore, it should be considered that the observed pattern reflects a treatment × duration relationship rather than a factorial design.

## Funding

No funding was received for this manuscript.

## Conflicts of Interest

The authors declare no conflicts of interest.

## Data Availability

All relevant data are within the article.
